# Trends in Mortality from External Causes Among Older Adults in Poland, 2000–2022: A Nationwide Joinpoint Regression Analysis by Sex and Age Group

**DOI:** 10.3390/medicina62101908

**Published:** 2026-10-02

**Authors:** Elżbieta Michalska, Małgorzata Pikala, Monika Burzyńska

**Affiliations:** 1 Department of Public Health, Epidemiology and Biostatistics, Public Health Observatory, Medical University of Lodz, Żeligowskiego 7/9, 90-752 Lodz, Poland; ejmichalska@gmail.com (E.M.); malgorzata.pikala@umed.lodz.pl (M.P.); 2 Successful Ageing Research Unit, Department of Internal Medicine and Geriatric Cardiology, Centre of Postgraduate Medical Education, 01-813 Warsaw, Poland

**Keywords:** ageing, mortality, external causes of death, falls, suicide, transport accidents, epidemiology, trends

## Abstract

*Background and Objectives*: External causes of death remain an important contributor to mortality among older adults. This study assessed long-term trends in mortality due to external causes, transport accidents, falls, and suicide among older adults in Poland between 2000 and 2022. *Materials and Methods*: Nationwide mortality data obtained from Statistics Poland were used to identify all deaths among individuals aged ≥65 years between 2000 and 2022 (*n* = 6,645,408). Mortality trends were analyzed separately for women and men aged 65–74 years and ≥75 years. Standardized death rates (SDRs) were calculated using the 2012 European Standard Population, and temporal trends were assessed using Joinpoint regression analysis. *Results*: Mortality due to transport accidents declined significantly in all age and sex groups. Among women aged 65–74 years, SDRs decreased from 46.6 to 28.1 per 100,000 for all external causes, while among men aged 75 years and older mortality due to transport accidents declined from 62.9 to 16.2 per 100,000. Falls became the leading external cause of death among adults aged ≥75 years and, in recent years, among men aged 65–74 years. Although fall-related mortality initially decreased, significant increases were observed among men aged ≥75 years after 2018. Suicide mortality remained substantially higher among men than women throughout the study period and exceeded mortality due to transport accidents among men for more than a decade. During the final years of observation, mortality due to all external causes increased among adults aged ≥75 years. *Conclusions*: The structure of external-cause mortality among older adults in Poland changed substantially between 2000 and 2022. While transport accident mortality markedly declined, falls emerged as the dominant external cause of death among the oldest-old, and suicide remained an important cause of mortality among older men. These findings support the implementation of targeted injury-prevention and mental health strategies in ageing populations.

## 1. Introduction

Population ageing is one of the most important demographic challenges worldwide. According to the World Health Organization (WHO), the number of people aged 60 years and older is expected to nearly double between 2015 and 2050, reaching approximately 2.1 billion individuals. As longevity increases, the burden of chronic diseases, disability, frailty, and injury-related health problems also rises, substantially affecting healthcare systems and social care services. Among older adults, external causes of death represent an important but often underappreciated contributor to mortality, disability, loss of independence, and reduced quality of life [1,2].

External causes of death comprise a heterogeneous group of conditions, including falls, transport accidents, intentional self-harm, and other injuries (ICD-10 codes V01–Y98). Globally, injuries account for more than 4 million deaths annually and remain a major public health challenge across all age groups. Older adults are particularly vulnerable to fatal injury outcomes because of frailty, multimorbidity, sarcopenia, impaired balance, sensory deficits, cognitive decline, and reduced physiological reserve. Consequently, injuries that may be survivable in younger age groups often result in severe disability or death among older individuals [1,3].

Among external causes, falls constitute one of the most important threats to healthy ageing. The WHO estimates that approximately 684,000 people die annually from falls worldwide, making falls the second leading cause of unintentional injury death globally. Adults aged 60 years and older have the highest risk of fatal falls, and approximately one-third of community-dwelling older adults experience at least one fall each year. Falls are associated with fractures, traumatic brain injuries, functional decline, institutionalization, and excess mortality. Furthermore, demographic projections suggest that the burden of falls will continue to increase as populations age [4,5,6].

Road traffic injuries remain another leading cause of injury-related mortality worldwide. Approximately 1.35 million people die each year as a result of road traffic crashes, while tens of millions experience non-fatal injuries. In the European Union, nearly half of all road traffic fatalities occur among individuals aged 65 years and older. Older pedestrians and cyclists constitute a particularly vulnerable group because age-related frailty substantially increases the risk of death following collision-related trauma. Although road safety has improved considerably over recent decades, population ageing has created new challenges for transport safety policies throughout Europe [7,8,9].

Suicide is another important external cause of death among older adults. In many high-income countries, suicide mortality is highest among older men. Chronic disease, disability, social isolation, bereavement, depression, and retirement-related transitions have all been identified as important determinants of suicidal behaviour in later life. The growing number of older adults worldwide has therefore increased interest in suicide prevention as an important component of healthy ageing strategies and public health policy [10,11,12].

Although falls, transport accidents, and suicides are recognized as major external causes of death among older adults, previous studies have usually examined these causes separately or analysed all individuals aged 65 years and older as a single population group. Older adults are not a homogeneous population, and the epidemiology of external-cause mortality may change substantially with advancing age. We therefore distinguished early old age (65–74 years) from later old age (≥75 years), consistent with previous population-based mortality studies in Poland, as the latter group is more likely to experience frailty, multimorbidity, functional limitations, and increased vulnerability to falls and other injury-related outcomes. This age stratification also allows potential differences in the relative contribution of transport accidents, falls, and suicide to be identified. Understanding long-term trends in mortality according to sex, age group, and specific categories of external causes may provide valuable evidence for public health planning and prevention programmes.

Therefore, the aim of this study was to assess trends in mortality due to external causes of death among older adults in Poland between 2000 and 2022, with particular emphasis on transport accidents, falls, and suicides. Analyses were performed separately for women and men in early old age (65–74 years) and late old age (≥75 years).

## 2. Materials and Methods

The study was based on a nationwide database containing information on all deaths among Polish residents aged 65 years and older between 2000 and 2022, obtained from Statistics Poland. The dataset included all registered deaths regardless of the place of death (hospital, home, or other settings), as recorded in the national death registry. In total, 6,645,408 deaths were included in the analysis.

The present study focused on mortality due to external causes of death (ICD-10 codes V01–Y98). Detailed analyses were performed for the three leading groups of external causes: transport accidents (V01–V99), falls (W00–W19), and intentional self-harm (suicide) (X60–X84). These causes were selected because they accounted for the largest proportion of deaths within the category of external causes among older adults.

Given well-established sex- and age-related differences in mortality patterns in later life, analyses were conducted separately for women and men in two age categories: early old age (65–74 years) and late old age (75 years and older). To evaluate changes in the mortality structure over time, proportional mortality ratios were calculated for the first (2000) and the last (2022) year of the study period. In addition, standardized death rates (SDRs) per 100,000 population were calculated.

Age standardization was performed using the direct method based on the revised 2012 European Standard Population (ESP) [13]. Temporal trends in SDRs were assessed using Joinpoint regression models implemented in the Joinpoint Regression Program developed by the U.S. National Cancer Institute within the Surveillance, Epidemiology, and End Results (SEER) Program [14]. Annual percent changes (APCs) were estimated for each identified trend segment, whereas average annual percent changes (AAPCs) were calculated for the entire study period. All estimates were presented with corresponding 95% confidence intervals (95% CIs). Statistical significance was assumed at *p* < 0.05.

The study protocol was approved by the Bioethics Committee of the Medical University of Lodz (approval No. RNN/422/12/KB). In accordance with national regulations and institutional requirements, informed consent was waived because the study was based exclusively on anonymized registry data.

## 3. Results

### 3.1. Distribution of External Causes of Death Among Older Adults

The leading categories of causes of death among the Polish population aged 65 years and older in 2022 were, in descending order, diseases of the circulatory system, malignant neoplasms, diseases of the respiratory system, diseases of the digestive system, and external causes. Regarding external causes, an increase in the proportion of deaths was observed among men in early old age (65–74 years), rising from 3.4% in 2000 to 3.7% in 2022. In the remaining groups, the proportion of deaths declined: among men aged 75 years and older, from 2.5% to 2.4%; among women aged 65–74 years, from 2.2% to 2.0%; and among women aged 75 years and older, from 2.7% to 2.1%.

The complete distribution of external causes of death is presented in Supplementary Tables S1 and S2. Across the four analysed sex- and age-specific groups, falls represented the largest proportion of external-cause deaths in 2022 (45.1%), followed by intentional self-harm (10.2%), other and unspecified accidental causes (8.8%), transport accidents (6.8%), complications of medical and surgical care (6.5%), accidental poisoning (5.3%), and events of undetermined intent (4.7%). Other causes included smoke, fire and flames (2.9%), other accidental threats to breathing (2.5%), forces of nature (1.8%), drowning and submersion (1.4%), and sequelae of external causes (1.4%), while the remaining categories each accounted for less than 1% of external-cause deaths. The relative contribution of individual causes differed substantially by sex and age group (Tables S1 and S2).

Within the category of external causes, the most frequent causes of death were transport accidents, falls, and suicides. The proportion of deaths due to transport accidents decreased in all analyzed groups. In contrast, mortality due to falls increased among both male age groups but decreased among both female age groups, whereas suicide mortality showed only minor changes across all analyzed groups.

**Figure 1:**
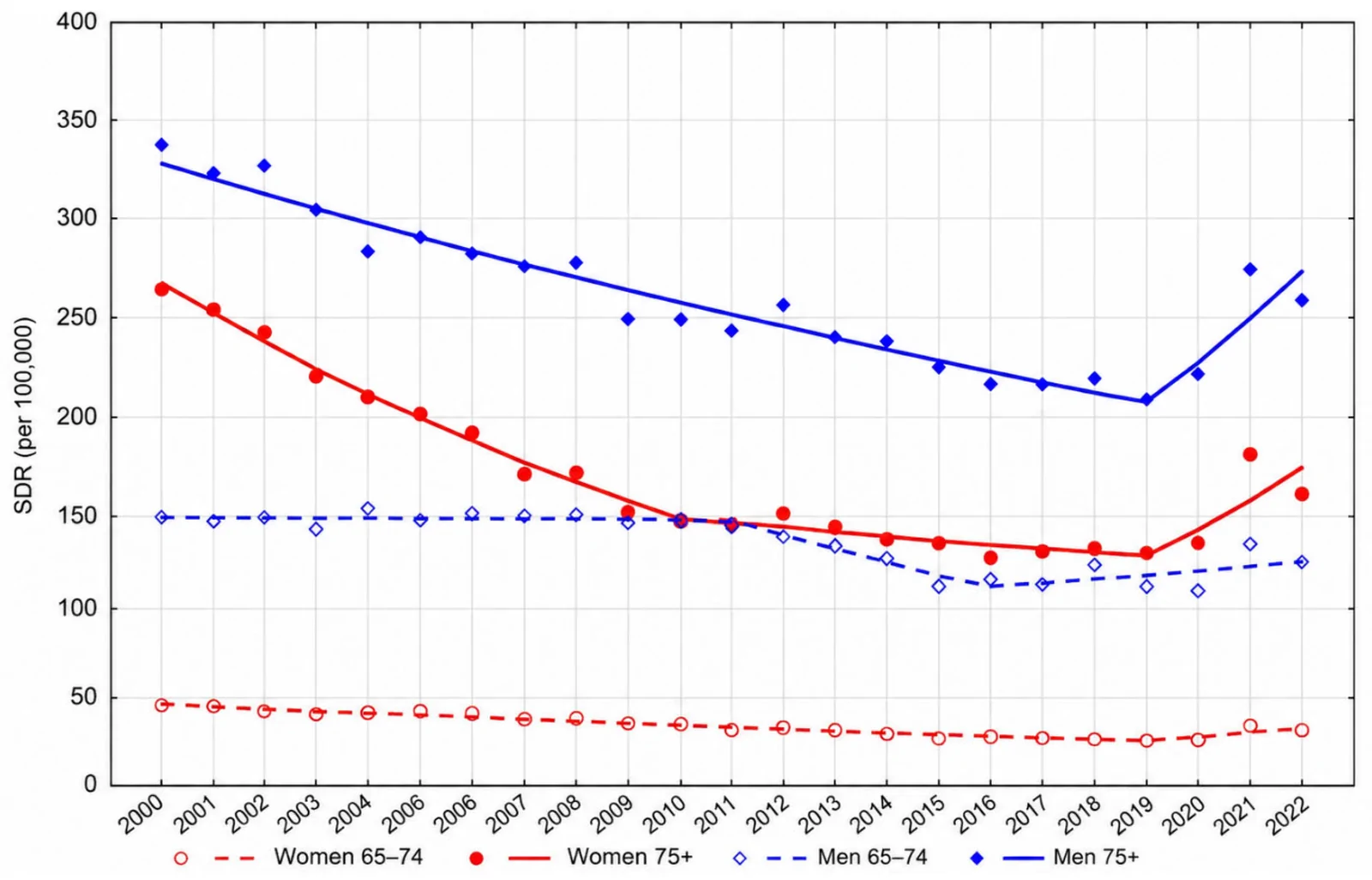
Trends in mortality due to external causes of death from 2000 to 2022 by sex and age groups of early and late old age.

Standardized death rates (SDRs) for external causes of death demonstrated an increase during the final years of the study period in all four subgroups. However, these increases were not statistically significant among women and men aged 65–74 years, whereas statistically significant upward trends were observed among both sexes aged 75 years and older (Figure 1).

### 3.2. Women Aged 65–74 Years

**Figure 2:**
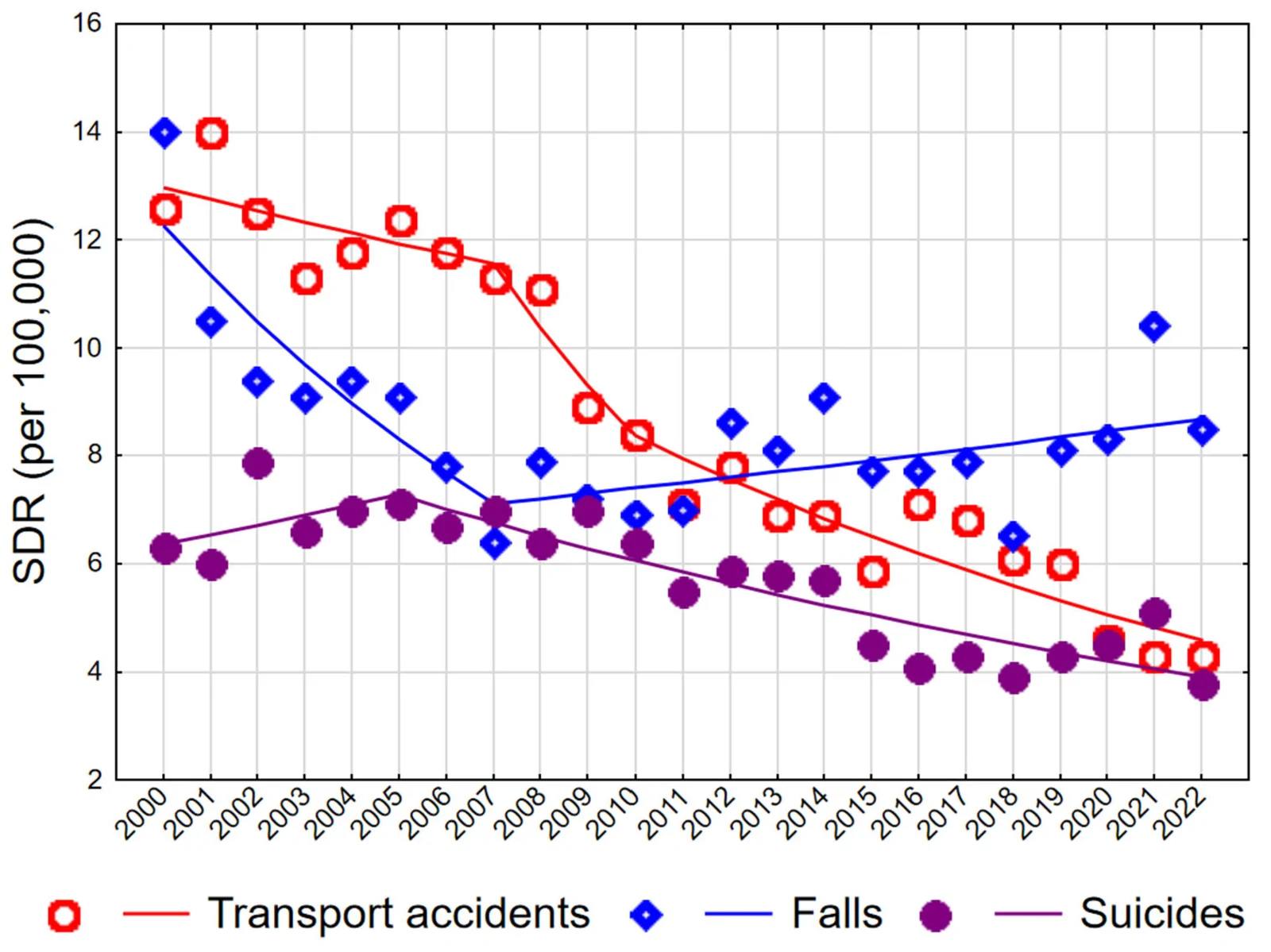
Trends in mortality from the leading external causes of death among women aged 65–74 years from 2000 to 2022.

Among women aged 65–74 years, a statistically significant decline in external-cause mortality was observed between 2000 and 2019 (APC = −2.9%, *p* < 0.05), with the standardized death rate decreasing from 46.6 to 28.1 per 100,000 population. Transport accidents represented the leading external cause during the initial years of the study period, whereas falls became the leading cause from 2012 onwards. Transport-related mortality declined significantly from 2010 onwards (APC = −4.9%, *p* < 0.05), while mortality due to falls showed a significant decline between 2000 and 2007, followed by a non-significant increasing tendency. Suicide mortality declined significantly from 2005 onwards (APC = −3.6%, *p* < 0.05) (Table 1, Figure 2).

### 3.3. Women Aged ≥75 Years

**Figure 3:**
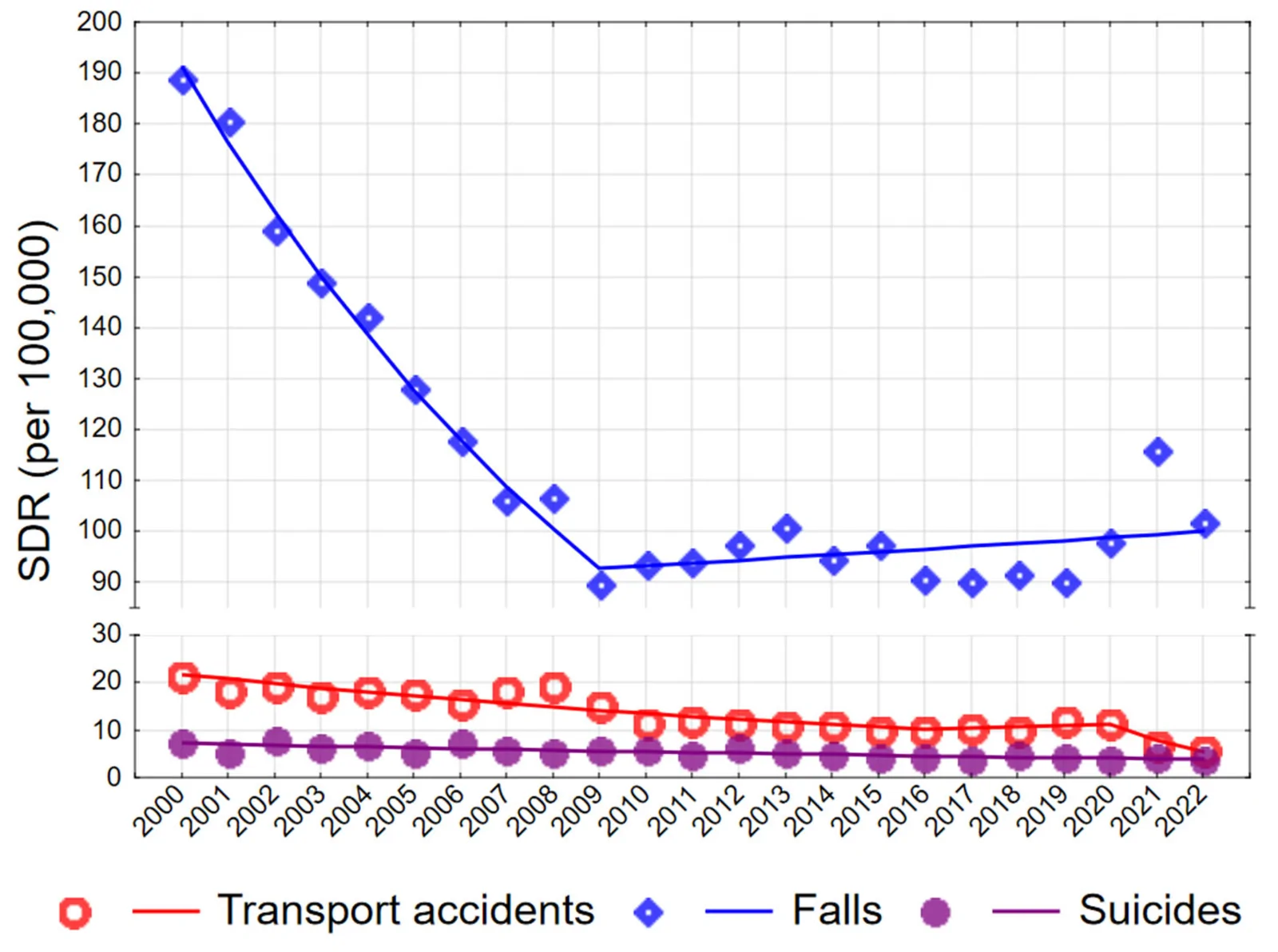
Trends in mortality from the leading external causes of death among women aged 75 years and over from 2000 to 2022.

Among women aged ≥75 years, falls were the dominant external cause throughout the study period. Fall-related mortality declined significantly between 2000 and 2009 (APC = −7.7%, *p* < 0.05), from 188.9 to 89.8 per 100,000, and subsequently remained relatively stable through 2022. Transport-related mortality declined significantly over the study period (AAPC = −6.0%, *p* < 0.05), from 21.5 per 100,000 in 2000 to 6.0 per 100,000 in 2022. Suicide mortality also decreased significantly (APC = −2.8%, *p* < 0.05) (Table 2, Figure 3).

### 3.4. Men Aged 65–74 Years

**Figure 4:**
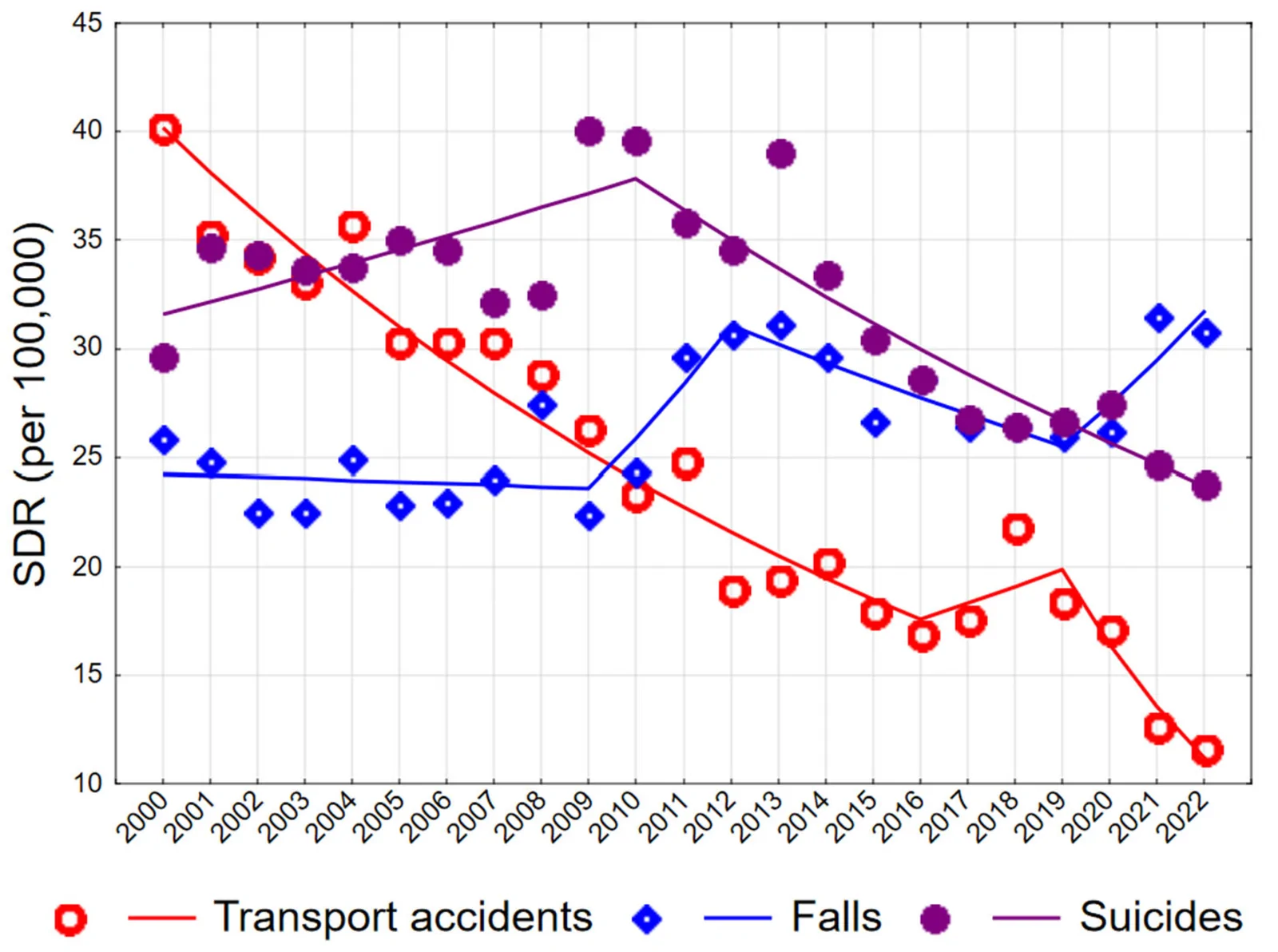
Trends in mortality from the leading external causes of death among men aged 65–74 years from 2000 to 2022.

Among men aged 65–74 years, transport accidents were the leading external cause during the early years of the study period, but their standardized mortality rate declined substantially from 40.2 per 100,000 in 2000 to 11.6 per 100,000 in 2022 (AAPC = −5.6%, *p* < 0.05). Suicide mortality was 29.6 per 100,000 in 2000 and 23.8 per 100,000 in 2022, with a significant increase between 2000 and 2010 followed by a significant decline from 2010 onwards. Falls became the leading external cause in 2021 and 2022 (Table 3, Figure 4).

### 3.5. Men Aged ≥75 Years

**Figure 5:**
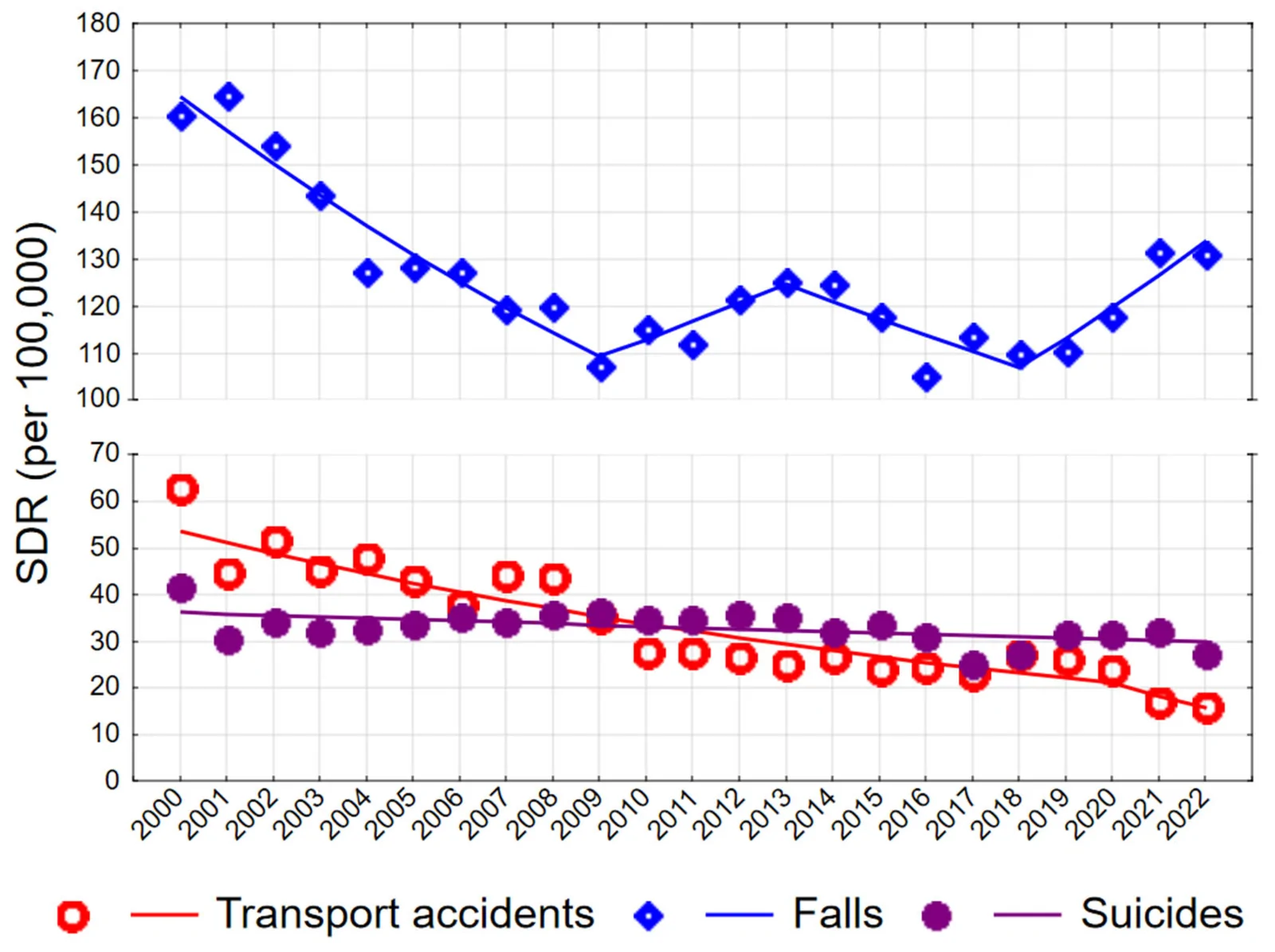
Trends in mortality from the leading external causes of death among men aged 75 years and over from 2000 to 2022.

Among men aged ≥75 years, falls were the most common external cause of death. Fall-related mortality declined from 160.6 per 100,000 in 2000 to 131.3 per 100,000 in 2022, with a significant increase observed only between 2018 and 2022 (APC = 5.7%, *p* < 0.05). Transport-related mortality declined substantially throughout the study period (AAPC = −5.4%, *p* < 0.05), from 62.9 to 16.2 per 100,000. Suicide mortality also declined significantly, from 41.7 to 27.6 per 100,000 (APC = −0.9%, *p* < 0.05) (Table 4, Figure 5).

## 4. Discussion

In this nationwide study, we observed a substantial overall decline in mortality due to external causes among older adults in Poland between 2000 and 2022, although the magnitude and direction of these changes varied considerably according to age, sex, and cause of death. The most pronounced reduction was observed for transport-related mortality, whereas mortality due to falls remained high and became increasingly prominent, particularly among adults aged 75 years and older. Among men, suicide represented an important and persistent cause of external-cause mortality and exceeded mortality due to transport accidents for more than a decade. These findings indicate that the structure of external-cause mortality among older adults has changed substantially over the study period. The relative contribution of falls increased in several groups, particularly among older men, despite declines or stabilization in fall-specific mortality rates. At the same time, transport-related mortality declined markedly, while suicide remained an important contributor to external-cause mortality among older men.

The marked decline in mortality due to transport accidents observed across all analysed groups is consistent with the long-term improvement in road safety documented in Poland. Road traffic injuries are the eighth leading cause of death worldwide, accounting for approximately 1.35 million deaths and 50 million injuries annually [15]. In Poland, approximately one in four deaths due to external causes is associated with transport accidents. Men are disproportionately affected, with mortality rates almost four times higher than those observed among women [16]. Traffic-related injuries are associated with high mortality and substantial morbidity, particularly due to severe head and hip injuries, which are especially common among older adults. According to Organisation for Economic Co-operation and Development (OECD) reports, approximately 45% of all road traffic fatalities in the European Union occur among older individuals [17]. Between 2011 and 2018, the number of fatal traffic accidents involving older adults increased by 3%, followed by a marked decline of 23% in 2020 compared with 2019. Among older traffic fatalities, 33% are pedestrians and 17% are cyclists. In several Central and Eastern European countries, pedestrians account for more than half of all road traffic deaths among older adults. The decline in mortality due to transport accidents observed in the present study may reflect long-term improvements in road safety implemented in Poland and across Europe during recent decades. Advances in vehicle safety technologies, better road infrastructure, stricter enforcement of traffic regulations, and improvements in trauma care have contributed to a substantial reduction in road traffic mortality. Nevertheless, older adults remain particularly vulnerable road users owing to increased physical frailty and a greater likelihood of sustaining severe injuries following collisions. European reports indicate that pedestrians and cyclists account for a considerable proportion of road traffic fatalities among older people, highlighting the importance of age-friendly transport policies and safer urban environments [7]. The decline observed in the study may have been partly influenced by the COVID-19 pandemic, as mobility restrictions and social distancing measures reduced traffic volume and consequently decreased both the number of road traffic accidents and accident-related injuries worldwide [18].

The present study also demonstrated that falls remain the leading external cause of death among both women and men aged 75 years and older. This finding is consistent with previous international studies [19,20,21] as well as results from the Polish PolSenior2 study [22,23]. Evidence from the literature suggests that most fall-related risk factors can be identified and that approximately one-third to two-thirds of falls may be preventable [24]. Older women with low body mass, reduced muscle strength, impaired gait, multimorbidity, polypharmacy (more than four medications daily), and a history of previous falls are particularly susceptible to falling [25]. Women are approximately three times more likely to experience a fall than men [26]. Nevertheless, men have a substantially higher risk of death following a fall, a pattern also observed in the present study in both early and late old age. This disparity may be explained by the higher burden of comorbidities among older men compared with women of the same age [27]. In addition, men experience falls under different circumstances and sustain different types of injuries. Falls among men occur more frequently outdoors and during acute episodes of illness and are more likely to result in traumatic injuries of the head and thorax, thereby increasing fall-related mortality [28,29,30].

The increasing importance of falls observed in the present study is consistent with demographic and epidemiological changes occurring across Europe. Population ageing has resulted in a growing number of individuals surviving to advanced ages, often with multimorbidity, frailty, sarcopenia, balance disorders, and functional limitations, all of which are recognized risk factors for falls and fall-related injuries. Consequently, the burden of falls is expected to increase further as the proportion of the oldest-old population continues to rise. According to the World Health Organization, falls are the second leading cause of unintentional injury deaths worldwide. Moreover, fall-related injuries constitute an important cause of disability, institutionalization, loss of independence, and healthcare expenditure among older adults [3]. Importantly, the increasing relative importance of falls should be distinguished from changes in fall-specific mortality rates. In women aged ≥75 years, falls accounted for 63.7% of external-cause deaths in 2022 compared with 66.3% in 2000, while the standardized mortality rate declined substantially from 188.9 to 102.1 per 100,000. Similarly, among men aged ≥75 years, the proportion of external-cause deaths attributable to falls increased from 41.3% to 49.3%, whereas the standardized mortality rate decreased from 160.6 to 131.3 per 100,000. Thus, the growing prominence of falls within the structure of external-cause mortality should not be interpreted uniformly as an increase in fall-related mortality rates. Rather, it partly reflects the substantially larger decline in other external causes, particularly transport-related mortality.

The findings of the present study have important implications for public health policy. Current international guidelines indicate that a substantial proportion of falls can be prevented through multifactorial interventions, including regular physical activity, strength and balance training, medication review, vision assessment, osteoporosis management, and home hazard reduction. Given the dominance of falls as a cause of mortality among adults aged 75 years and older, implementation of evidence-based fall prevention programmes should become an integral component of healthy ageing strategies and geriatric care in Poland [4].

Another noteworthy observation is that, in Poland, mortality due to suicide among men has exceeded mortality due to transport accidents for more than a decade. Similar findings have been reported in other countries, including Australia, where suicide accounts for more deaths among men than road traffic accidents [31]. In Australia, suicide rates increased substantially between 2001 and 2021 among men aged 55–74 years and women aged 55–84 years. Among older men, suicide rates increased progressively with age and reached their highest levels in those aged 85 years and older. According to data from the U.S. Centers for Disease Control and Prevention (CDC), nearly 53% of suicide deaths recorded in the United States in 2022 occurred among individuals aged 45 years and older. Moreover, in 2021 suicide mortality rates were considerably higher among men than women in age groups above 55 years [32].

The elevated suicide mortality observed among older adults may be partly attributable to age-related deterioration in health status, increasing multimorbidity, and the consequent rise in the prevalence of mental health disorders that predispose individuals to suicidal behaviour [33]. Retirement has also been identified as an important life transition associated with heightened suicide risk. Furthermore, studies indicate that older adults frequently do not communicate their suicidal intentions and are more likely to attempt suicide in circumstances that preclude timely intervention, a phenomenon particularly pronounced among men [34].

The observed sex differences in suicide mortality are consistent with findings reported from other countries. Although depressive symptoms and suicidal ideation are common among both sexes, older men are more likely to die by suicide because they tend to use more lethal methods and are less likely to seek professional help for mental health problems. Furthermore, social isolation, bereavement, retirement, chronic diseases, disability, and loss of functional independence have all been identified as important determinants of suicide risk in later life. These findings emphasize the need for targeted suicide prevention programmes, including systematic screening for depression and loneliness in primary care and geriatric settings.

The increase in external-cause mortality observed during the final years of the study period, particularly among adults aged ≥75 years, should be interpreted cautiously. The COVID-19 pandemic may have influenced mortality patterns through changes in mobility, healthcare access, physical activity, and social isolation; however, the registry-based design of the present study does not allow the observed changes to be directly attributed to the pandemic. Therefore, the contribution of COVID-19-related factors to the observed temporal patterns remains uncertain.

Taken together, these findings indicate a shift in the structure of external-cause mortality among older adults in Poland. While mortality from transport accidents declined substantially over the study period, falls became increasingly prominent, particularly among adults aged 75 years and older and, in the final years, among men aged 65–74 years. At the same time, suicide remained a disproportionately important cause of death among older men. These divergent trajectories suggest that prevention strategies for older adults should not be uniform but should be tailored according to age, sex, and the specific external cause of death.

To our knowledge, few nationwide studies have comprehensively examined long-term trends in mortality due to external causes among older adults in Poland using joinpoint regression analysis. By examining mortality separately according to sex and age group, the study revealed substantial heterogeneity in mortality patterns that would remain obscured in analyses limited to the older population as a whole. These findings contribute to a better understanding of injury-related mortality in ageing societies and provide evidence that may support the development of targeted preventive interventions and public health policies.

### Limitations

Several limitations should be acknowledged. First, the study relied on routinely collected national mortality statistics and therefore depended on the accuracy of cause-of-death certification and ICD-10 coding. Misclassification of some external causes of death cannot be completely excluded. In addition, changes over time in death certification practices and coding procedures may have influenced the classification of specific external causes of death and should be considered when interpreting long-term trends, particularly for individual categories such as falls. Second, the ecological and observational nature of the analysis precludes causal inference regarding the factors underlying the observed mortality trends. Third, the database did not contain information on socioeconomic status, educational attainment, place of residence, health behaviours, functional status, frailty, or comorbidities, which may partially explain the observed mortality patterns. Nevertheless, the major strengths of the study include its nationwide coverage, inclusion of all registered deaths in Poland, and a long observation period spanning 23 years.

## 5. Conclusions

Mortality due to external causes among older adults in Poland decreased between 2000 and 2022, although the patterns varied according to sex and age. Transport accident mortality declined substantially in all analysed groups. Falls remained the leading external cause of death among adults aged 75 years and older and became the most common external cause of death among men aged 65–74 years in the final years of the study period. Mortality due to suicide was consistently higher among men than women and exceeded mortality due to transport accidents among men for more than a decade. These findings indicate the need for targeted prevention strategies addressing falls among the oldest-old population and suicide among older men.

## Figures and Tables

**Table 1:** Temporal trends in standardized death rates (SDRs) for the most common causes of death among women aged 65–74 years between 2000 and 2022: a joinpoint regression analysis.

Death Cause	Number of Joinpoints	Years	APC (95% CI)	AAPC (95% CI)
External causes of death (V01–Y98):	1	2000–2019	−2.9 * (−3.3; −2.5)	−1.6 * (−2.5; −0.6)
		2019–2022	7.5 (−0.1; 15.7)	
Transport accidents (V01–V99)	2	2000–2007	−1.6 (−5.3; 2.2)	−4.6 * (−8.2; −0.9)
		2007–2010	−10.2 (−32.3; 19.1)	
		2010–2022	−4.9 * (−6.5; −3.3)	
Falls (W00–W19)	1	2000–2007	−7.5 * (−11.3; −3.5)	−1.6 * (−3.0; 0.0)
		2007–2022	1.3 (0.0; 2.7)	
Suicides (X60–X84)	1	2000–2005	2.7 (−4.3; 10.2)	−2.2 * (−3.8; −0.5)
		2005–2022	−3.6 * (−4.6; −2.5)	

* *p* < 0.05.

**Table 2:** Temporal trends in standardized death rates (SDRs) for the most common causes of death among women aged 75 years and over between 2000 and 2022: a joinpoint regression analysis.

Death Cause	Number of Joinpoints	Years	APC (95% CI)	AAPC (95% CI)
External causes of death (V01–Y98):	2	2000–2010	−5.7 * (−6.8; −4.6)	−1.9 * (−3.1; −0.7)
		2010–2019	−1.5 (−3.1; 0.2)	
		2019–2022	10.3 * (2.4; 18.8)	
Transport accidents (V01–V99)	2	2000–2016	−4.7 * (−5.8; −3.5)	−6.0 * (−9.5; −2.3)
		2016–2020	2.9 (−12.1; 20.3)	
		2020–2022	−29.6 * (−48.5; −3.6)	
Falls (W00–W19)	1	2000–2009	−7.7 * (−9.1; −6.4)	−2.9 * (−3.6; −2.2)
		2009–2022	0.6 (−0.3; 1.5)	
Suicides (X60–X84)	0	2000–2022	−2.8 * (−3.5; −2.1)	

* *p* < 0.05.

**Table 3:** Temporal trends in standardized death rates (SDRs) for the most common causes of death among men aged 65–74 years between 2000 and 2022: a joinpoint regression analysis.

Death Cause	Number of Joinpoints	Years	APC (95% CI)	AAPC (95% CI)
External causes of death (V01–Y98):	2	2000–2011	0.1 (−0.9; 0.8)	−0.7 (−1.8; 0.4)
		2011–2016	−5.1 * (−8.9; −1.1)	
		2016–2022	1.8 (−0.4; 4.0)	
Transport accidents (V01–V99)	2	2000–2016	−5.0 * (−5.8; −4.3)	−5.6 * (−8.4; −2.8)
		2016–2019	4.3 (−15.4; 28.6)	
		2019–2022	−17.4 * (−25.6; −8.2)	
Falls (W00–W19)	3	2000–2009	−0.3 (−2.0; 1.4)	1.2 (−1.8; 4.3)
		2009–2012	9.5 (−9.4; 32.3)	
		2012–2019	−2.7 * (−5.1; −0.2)	
		2019–2022	7.5 (−2.8; 19.0)	
Suicides (X60–X84)	1	2000–2010	1.8 * (0.3; 3.4)	−1.3 * (−2.2; −0.4)
		2010–2022	−3.8 * (−4.9; −2.7)	

* *p* < 0.05.

**Table 4:** Temporal trends in standardized death rates (SDRs) for the most common causes of death among men aged 75 years and over between 2000 and 2022: a joinpoint regression analysis.

Death Cause	Number of Joinpoints	Years	APC (95% CI)	AAPC (95% CI)
External causes of death (V01–Y98):	1	2000–2019	−2.4 * (−2.7; −2.0)	−0.8 * (−1.6; 0.0)
		2019–2022	9.6 * (3.3; 16.3)	
Transport accidents (V01–V99)	1	2000–2020	−4.6 * (−5.5; −3.6)	−5.4 * (−8.4; −2.3)
		2020–2022	−13.6 (−40.2; 24.8)	
Falls (W00–W19)	3	2000–2009	−4.4 * (−5.6; −3.2)	−0.9 (−2.6; 0.7)
		2009–2013	3.4 (−3.4; 10.6)	
		2013–2018	−3.0 (−7.1; 1.3)	
		2018–2022	5.7 * (1.3; 10.4)	
Suicides (X60–X84)	0	2000–2019	−0.9 * (−1.4; −0.3)	

* *p* < 0.05.

## Data Availability

The data presented in this study are available on request from the corresponding author.

## Supplementary Materials

The following supporting information can be downloaded at https://www.mdpi.com/article/10.3390/medicina62101908/s1, Table S1. Number and percentage of deaths from external causes among women aged 65–74 and ≥75 years in 2000 and 2022. Table S2. Number and percentage of deaths from external causes among men aged 65–74 and ≥75 years in 2000 and 2022.
