# Supplementary material for: Trends in Mortality from External Causes Among Older Adults in Poland, 2000–2022: A Nationwide Joinpoint Regression Analysis by Sex and Age Group
Source: Medicina (Kaunas). 2026 Oct 2;62(10):1908. doi: 10.3390/medicina62101908 (PMC13707054; doi:10.3390/medicina62101908)
Supplement: Supplementary file 1 [file medicina-62-01908-s001.zip › medicina-4536426-supplementary.pdf]

Supplementary Table S1. Number and percentage of deaths from external causes among women aged 65–74 and ≥75 years in 2000 and 2022

External causes of mortality	2000				2022			
	65-74		75+		65-74		75+	
	Number	%	Number	%	Number	%	Number	%
Transport accidents (V01-V99)	218	26.7	256	9.3	110	13.3	112	3.5
Falls (W00-W19)	240	29.4	1831	66.3	217	26.2	2036	63.7
Exposure to inanimate mechanical forces (W20-W49)	7	0.9	1	0.0	4	0.5	13	0.4
Exposure to animate mechanical forces (W50-W64)	4	0.5	11	0.4	3	0.4	1	0.0
Accidental drowning and submersion (W65-W74)	27	3.3	32	1.2	17	2.1	19	0.6
Other accidental threats to breathing (W75-W84)	12	1.5	15	0.5	33	4.0	69	2.2
Exposure to electric current, radiation and extreme ambient air temperature and pressure (W85-W99)	1	0.1	3	0.1	1	0.1	3	0.1
Exposure to smoke, fire and flames (X00-X09)	15	1.8	49	1.8	33	4.0	64	2.0
Contact with heat and hot substances (X10-X19)	3	0.4	10	0.4	2	0.2	8	0.3
Contact with venomous animals and plants (X20-X29)	0	0.0	1	0.0	0	0.0	0	0.0
Exposure to forces of nature (X30-X39)	3	0.4	17	0.6	14	1.7	22	0.7
Accidental poisoning by and exposure to noxious substances (X40-X49)	23	2.8	37	1.3	60	7.3	49	1.5
Overexertion, travel and privation (X50-X57)	0	0.0	4	0.1	2	0.2	9	0.3
Accidental exposure to other and unspecified factors (X58-X59)	47	5.8	205	7.4	48	5.8	353	11.0
Intentional self-harm (X60-X84)	109	13.4	93	3.4	99	12.0	73	2.3
Assault (X85-Y09)	17	2.1	22	0.8	10	1.2	9	0.3
Event of undetermined intent (Y10-Y34)	66	8.1	129	4.7	60	7.3	84	2.6
Legal intervention and operations of war (Y35-Y36)	1	0.1	0	0.0	0	0.0	0	0.0
Complications of medical and surgical care (Y40-Y84)	18	2.2	34	1.2	94	11.4	223	7.0
Sequelae of external causes of morbidity and mortality (Y85-Y89)	4	0.5	11	0.4	20	2.4	51	1.6
TOTAL	815	100.0	2761	100.0	827	100.0	3198	100.0

Supplementary Table S2. Number and percentage of deaths from external causes among men aged 65–74 and ≥75 years in 2000 and 2022

External causes of mortality	2000				2022			
	65-74		75+		65-74		75+	
	Number	%	Number	%	Number	%	Number	%
Transport accidents (V01-V99)	490	26.5	315	19.5	234	9.2	151	6.4
Falls (W00-W19)	310	16.8	667	41.3	612	24.1	1156	49.3
Exposure to inanimate mechanical forces (W20-W49)	18	1.0	8	0.5	37	1.5	31	1.3
Exposure to animate mechanical forces (W50-W64)	10	0.5	6	0.4	11	0.4	2	0.1
Accidental drowning and submersion (W65-W74)	47	2.5	25	1.5	70	2.8	22	0.9
Other accidental threats to breathing (W75-W84)	47	2.5	25	1.5	58	2.3	62	2.6
Exposure to electric current, radiation and extreme ambient air temperature and pressure (W85-W99)	10	0.5	1	0.1	10	0.4	10	0.4
Exposure to smoke, fire and flames (X00-X09)	43	2.3	33	2.0	113	4.4	48	2.0
Contact with heat and hot substances (X10-X19)	13	0.7	12	0.7	8	0.3	6	0.3
Contact with venomous animals and plants (X20-X29)	0	0.0	1	0.1	1	0.0	0	0.0
Exposure to forces of nature (X30-X39)	26	1.4	10	0.6	98	3.9	28	1.2
Accidental poisoning by and exposure to noxious substances (X40-X49)	81	4.4	30	1.9	305	12.0	61	2.6
Overexertion, travel and privation (X50-X57)	0	0.0	0	0.0	11	0.4	5	0.2
Accidental exposure to other and unspecified factors (X58-X59)	80	4.3	77	4.8	166	6.5	220	9.4
Intentional self-harm (X60-X84)	363	19.6	215	13.3	475	18.7	261	11.1
Assault (X85-Y09)	54	2.9	22	1.4	16	0.6	7	0.3
Event of undetermined intent (Y10-Y34)	205	11.1	124	7.7	165	6.5	109	4.7
Legal intervention and operations of war (Y35-Y36)	0	0.0	0	0.0	0	0.0	0	0.0
Complications of medical and surgical care (Y40-Y84)	41	2.2	31	1.9	117	4.6	145	6.2
Sequelae of external causes of morbidity and mortality (Y85-Y89)	11	0.6	14	0.9	36	1.4	20	0.9
TOTAL	1849	100.0	1616	100.0	2543	100.0	2344	100.0
